# Slow‐growing cervical mass in a young woman

**DOI:** 10.1002/ccr3.2856

**Published:** 2020-04-14

**Authors:** Sabrina De Stefano, Michele Sessa, Domenico Cuda

**Affiliations:** ^1^ Department of Otorhinolaryngology and Otoneurosurgery University Hospital of Parma Parma Italy; ^2^ Department of Otorhinolaryngology 'Guglielmo da Saliceto' Hospital Piacenza Italy

**Keywords:** cervical mass, critical care medicine, ear, nose and throat

## Abstract

ENT specialists should never underestimate the clinical benign course of cervical masses both in adults and in young patients.

## Graphical abstract

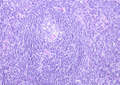

## 1 CASE PRESENTATION

Castleman disease is a rare lymphoproliferative disorder and mostly manifests as unicentric disease with an enlarged lymph nodal mass. Lymph nodes affected by Castleman disease can mimic both benign and malignant disorders of the neck. Surgery can provide histopathologic diagnosis and cure with good prognosis for unicentric Castleman disease.

**FIGURE 1:**
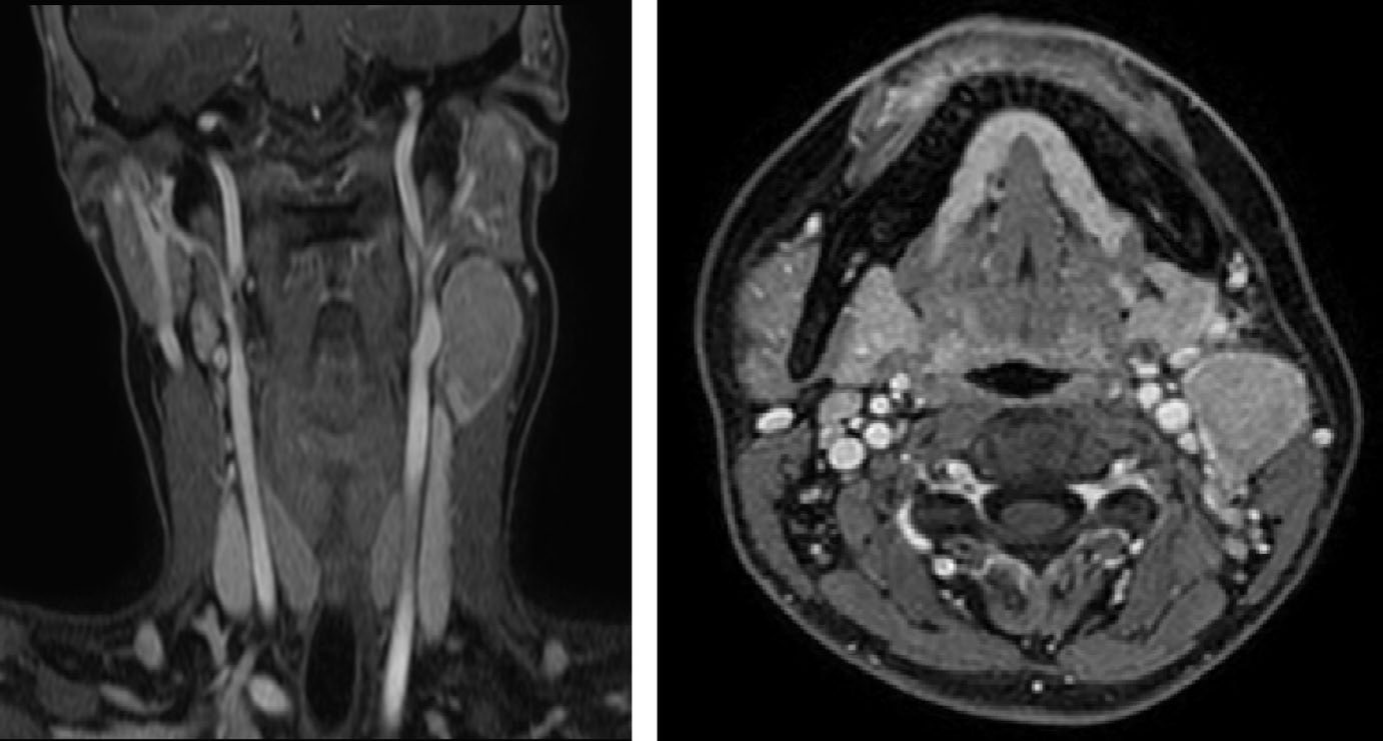
MRI coronal and axial scans show an ovoid mass with peripheral contrast enhancement causing displacement of surrounding musculature and vascular structures

**FIGURE 2:**
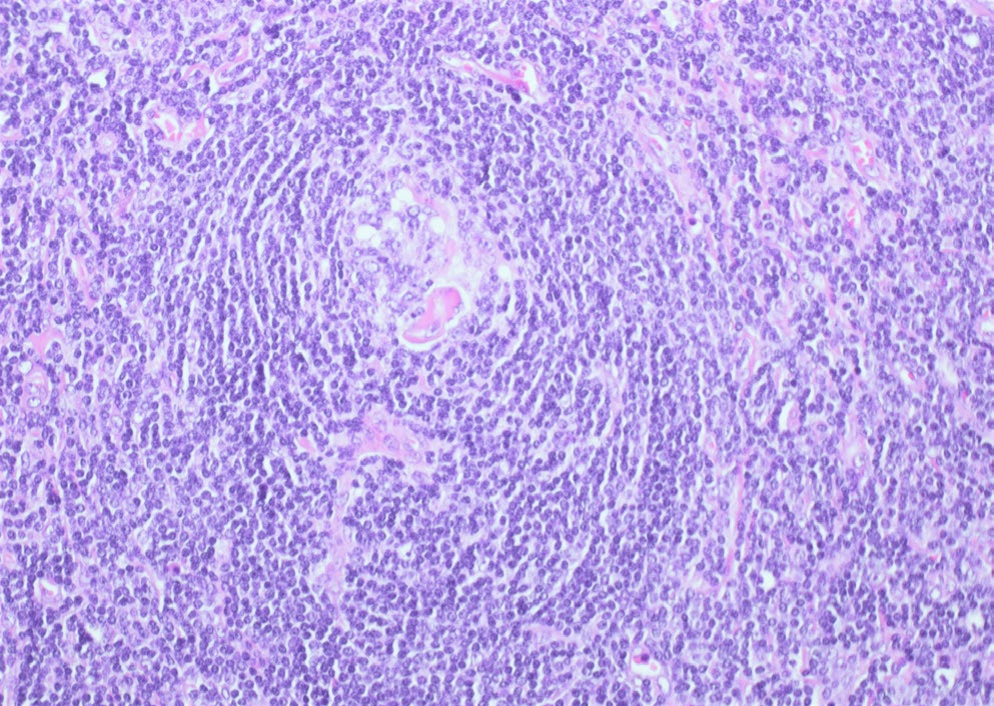
Penetrating vessel with hyalinized wall in the germinal center of the follicle (H&E 40x)

**FIGURE 3:**
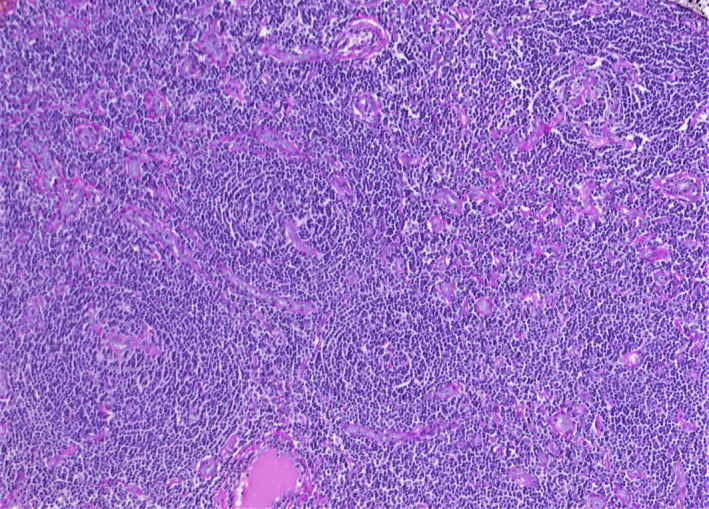
Follicular structures with thick mantle zones. Blood vessels with sclerotic walls are present in the interfollicular areas (H&E 20x)

A 20 years‐old woman referred to our evaluation for an asymptomatic slow‐growing cervical mass. The patient complained about the discomfort related to the esthetic appearance of the mass, and her past medical history was unremarkable. Magnetic resonance imaging (MRI) revealed an ovoid mass with peripheral contrast enhancement (Figure 1). The patient underwent surgery for excision. The histopathologic diagnosis was Castleman disease (CD). CD is a rare lymphoproliferative disorder, and the most common locations are mediastinum (60%), neck (14%), and abdomen (11%). The morphologic classification distinguishes between unicentric or multicentric CD. Unicentric CD shows a localized lymph node involvement, and multicentric CD is characterized by disseminated lymphadenopathies. The histologic patterns are hyaline vascular type, plasma cell type, and mixed type. The hyaline vascular type's histologic features are small atrophic germinal centers of the lymphoid follicles, lymphocyte depletions, and radially penetrating capillaries with hyaline deposits, creating a so‐called image of lollipop follicles (Figure 2). The mantle zone is characterized by a concentric layer of lymphocytes, and interfollicular areas are composed by proliferation of vessels with sclerotic walls (Figure 3).^1^ The radiological findings are nonspecific.^2^ In the setting of confirmed CD, the patient undergoes clinical peripheral lymph node examination, but in case of any clinical suspicion ultrasonography or whole‐body contrast computed tomographic scan can be performed to check out for more affected lymph nodes. CD should be considered in the differential diagnosis of slow‐growing cervical masses along with lymphomas, metastatic adenopathy, and infectious or inflammatory causes of adenopathy.

## CONFLICT OF INTEREST

The authors declare that there is no conflict of interest regarding the publication of this article.

## AUTHOR CONTRIBUTIONS

All authors contributed to the care of this patient. The authors wrote and critically reviewed the manuscript.

## Data Availability

All data underlying the results are available as part of the article, and no additional source data are required.
